# Ecological relevance of skeletal fatty acid concentration and composition in Mediterranean scleractinian corals

**DOI:** 10.1038/s41598-017-02034-2

**Published:** 2017-05-16

**Authors:** Chiara Samorì, Erik Caroselli, Fiorella Prada, Michela Reggi, Simona Fermani, Zvy Dubinsky, Stefano Goffredo, Giuseppe Falini

**Affiliations:** 1 Department of Chemistry ‘Giacomo Ciamician’, University of Bologna, via Selmi 2, 40126 Bologna, Italy; 2 Marine Science Group, Department of Biological, Geological and Environmental Sciences, University of Bologna, via Selmi 3, 40126 Bologna, Italy; 3 The Mina & Everard Goodman Faculty of Life Sciences, Bar-Ilan University, Ramat-Gan, 5290002 Israel

## Abstract

The intra-skeletal fatty acid concentration and composition of four Mediterranean coral species, namely *Cladocora caespitosa*, *Balanophyllia europaea*, *Astroides calycularis* and *Leptopsammia pruvoti*, were examined in young and old individuals living in three different locations of the Mediterranean Sea. These species are characterized by diverse levels of organization (solitary or colonial) and trophic strategies (symbiotic or non-symbiotic). Fatty acids have manifold fundamental roles comprehensive of membrane structure fluidity, cell signaling and energy storage. For all species, except for *B*. *europaea*, the intra-skeletal fatty acid concentration was significantly higher in young individuals than in old ones. Moreover, fatty acid concentration was higher in colonial corals than in solitary ones and in the symbiotic corals compared to non-symbiotic ones. Analysis by gas chromatography-mass spectrometry (GC-MS) revealed that palmitic acid (16:0) was the most abundant fatty acid, followed by stearic (18:0) in order of concentration. Oleic acid (18:1) was detected as the third main component only in skeletons from symbiotic corals. These results suggest that, in the limits of the studied species, intra-skeletal fatty acid composition and concentration may be used for specific cases as a proxy of level of organization and trophic strategy, and eventually coral age.

## Introduction

Lipids are important energy reserves, mainly stored in the animal tissue as wax esters and triglycerides, or in the membranes as sterols and polyunsaturated fatty acids. Since lipid composition is often specific to particular groups of organisms^1^, the analysis of lipids, such as fatty acids (FAs), gives useful information on their autotrophic or heterotrophic origin^2^. Indeed, FAs structure and production are dependent upon lipogenesis pathways and associated enzymes, which change among organisms. In addition, the absence of certain functional lipogenesis pathways can be compensated by obtaining FAs from dietary sources^3, 4^.

In corals, the bulk of photosynthetically fixed carbon is translocated from the dinoflagellate symbionts to the coral host, providing it with up to 95% of its daily metabolic energy requirements^5–8^. Excess fixed carbon is stored in the host tissue as lipids, representing significant energy reserves^4^.

In scleractinian corals, polar lipids and sterols are the structural basis of symbiotic zooxanthellae (i.e., endosymbiotic dinoflagellates of the Symbiodinium group^8^) cell membranes, while triacylglycerols, wax, and sterol esters serve as storage lipids and determine the energy balance of the animal^4^.

In scleractinian corals lipids are found in the soft tissue and the skeleton, representing about 10–30% (*w*/*w*) of dry soft tissue weight^5, 9, 10^ and tenths of a percentage (*w*/*w*) of the aragonitic skeleton^11^.

The concentration of total lipids in coral tissues varies with the season, depth, illumination, and other environmental factors. The main cause of the dependence of lipids on light intensity is the change of their biosynthesis in symbiotic zooxanthellae^8, 9^. Seasonal changes of FAs in reef-building corals have been reported for a number of Caribbean^10, 12^, Red Sea^12–14^, Japanese^15^, and Hawaiian corals^16^. Moreover, lipid and FA composition of a coral colony are related to the mode of nutrition and may be impacted by environmental conditions^15^. Grottoli *et al*.^4^ observed a decrease in total lipid abundance in bleached *Porites compressa* corals, but no change in *Montipora verrucosa*. Bachok *et al*.^17^ observed a marked decrease in lipid concentration and the total FA component, particularly polyunsaturated FAs, in bleached *Pavona frondifera* corals compared to healthy nearby specimens. In most species, heterotrophy increases protein and/or lipids concentration in coral tissue^18–21^.

The preservation of lipids in aragonitic coral skeletons has been demonstrated, providing the potential for construction of paleoenvironmental records^22^. The FAs and sterols in these skeletons are more consistent with the lipidic component of eukaryotic cell walls and probably represent invertebrate cellular material trapped during mineralization^23–26^. In this view, the FAs can be potentially used as proxies due to the advantage of being preserved and trapped within the skeleton mineralization, taking into account that coral skeletons do not undergo a dissolution-reprecipitation process^27, 28^.

**Figure 1:**
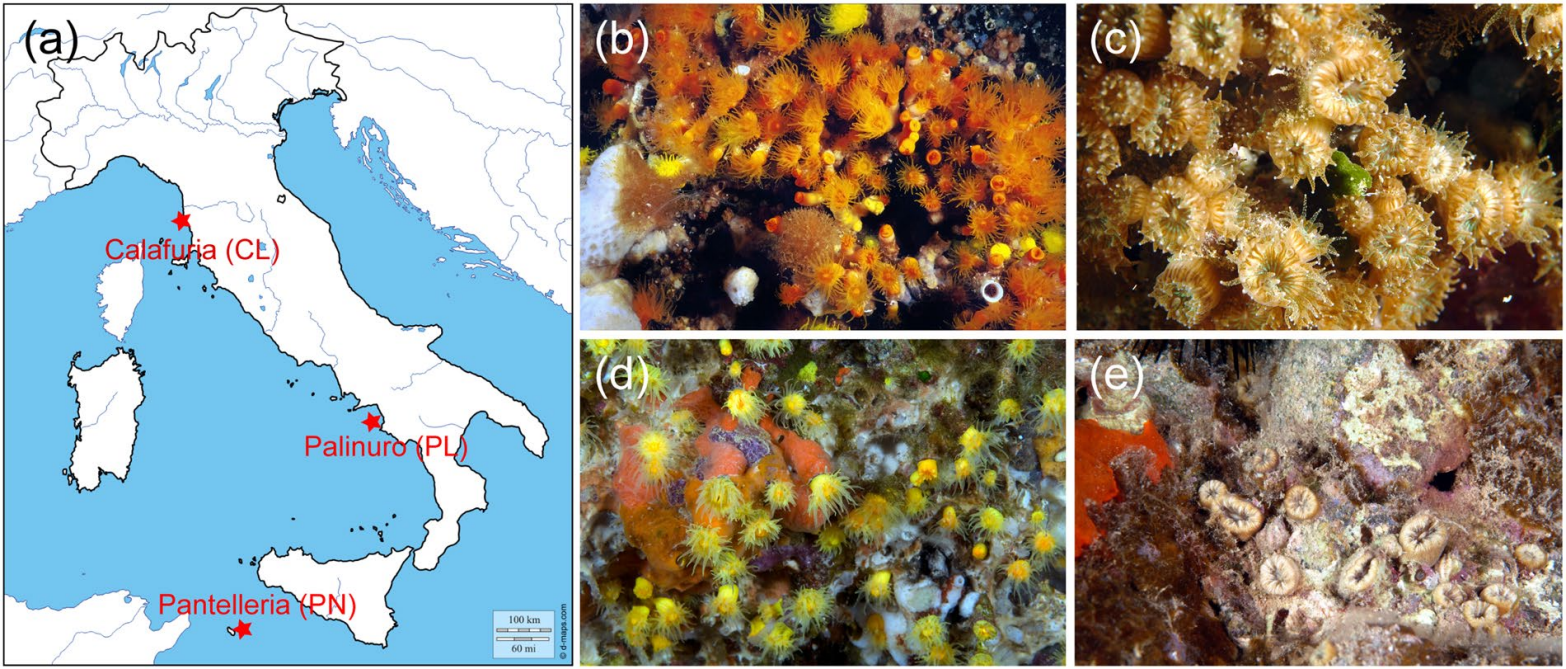
Collection sites and species. (**a**) Map of Italy showing the sites where corals were collected: Pantelleria island (PN), Palinuro (PL) and Calafuria (CL). (**b**–**e**) *In situ* camera pictures of the studied species: (**b**) *A*. *calycularis*, (**c**) *C*. *caespitosa*, (**d**) *L*. *pruvoti* and (**e**) *B*. *europaea*. The map of Italy was downloaded from the web site http://d-maps.com/carte.php?num_car=2332&lang=en (no permission required) and labeled using Microsoft Office PowerPoint 2007 (University of Bologna license). The camera pictures (**b**–**e**) are used with the permission of the photographer Gianni Neto.

This is the first study exploring the intra-skeletal FA content, speciation and variation with age in four Mediterranean coral species, namely the colonial symbiotic coral *Cladocora caespitosa*, the solitary symbiotic *Balanophyllia europaea*, the colonial non-symbiotic *Astroides calycularis* and the solitary non-symbiotic *Leptopsammia pruvoti*, living in three different locations in the Mediterranean Sea (Fig. 1).

## Materials and Methods

### Organism collection and soft tissue removal

Specimens of *C*. *caespitosa* (CCA), *B*. *europaea* (BEU), *A*. *calycularis* (ACL) and *L*. *pruvoti* (LPR), were randomly collected by SCUBA diving between July 1^st^ 2010 and March 24^th^ 2011, from three sites: Calafuria (CL), Palinuro (PL), and Pantelleria Island (PN) (Fig. 1). As reported in previous studies^29, 30^, these three locations are characterized by different average annual sea surface temperatures (SST) and solar radiation (SR) values, with an increase of almost 2 °C in SST and 44 W m^−2^ in SR going from CL to PN. At each site, corals of different sizes were sampled, to have a minimum of five individuals for each of the two selected size (polyp length: maximum diameter of the oral disk) classes for the four species. The two size classes represented two vital stages with respect to sexual maturity^31, 32^: the class of young individual polyp (before sexual maturity: *B*. *europaea* 0–5 mm; *L*. *pruvoti* 0–3 mm; *A*. *calycularis* and *C*. *caespitosa* 0–3 mm) and old individual polyp (after sexual maturity: *B*. *europaea* >10 mm; *L*. *pruvoti* >6 mm; *A*. *calycularis* and *C*. *caespitosa* >6 mm). Size at sexual maturity in *C*. *caespitosa* is not known so the same size as in the colonial *A*. *calycularis* was assumed. The samples were manipulated and handled with gloves to avoid human lipid contaminations.

The polyps for each species were decomposed by a 3 h immersion in distilled water that destroyed the cells. Removal of the remaining living tissues was done by using a water-jet, followed by two sequential immersions in 3% (*v*/*v*) NaClO under gentle shacking for 24 h to remove organic contaminants and then sonicated for 5 min; the latter ensured the complete cleanness of the samples. Then the samples were rinsed with Milli-Q water (18.2 MΩ·cm at 25 °C) and air-dried. Skeleton samples were collected from the oral region of the skeleton breaking mechanically fragments of septa. The skeleton fragments were then ground to powder in an agata mortar. Three samples were collected and ground for each skeleton.

### Fatty acid extraction and analysis

Powder of coral skeleton samples (about 50 mg) were extracted under reflux with chloroform/methanol mixture (2:1 *v/v*, 4 mL) for 1.5 h; the solvent phase was then removed and the procedure was repeated three times. The solvent phases were collected and concentrated by evaporation. The total FA content was determined as follows: the lipid extracts were dissolved in dimethylcarbonate (0.4 mL), 2,2-dimethoxypropane (0.1 mL) and 0.5 M NaOH in MeOH (0.1 mL), and then placed in an incubator at 90 °C for 30 min. After cooling for 5 min to room temperature, 1.3 M BF_3_-methanol 10% (w/w) reagent (0.7 mL) was added before repeating the incubation for 30 min. After cooling for 5 min to room temperature, saturated NaCl aqueous solution (2 mL) and hexane (1 mL) containing methyl nonadecanoate (20 μg) were added and the samples were centrifuged at 4000 rpm for 1 min. The upper hexane-dimethylcarbonate layer, containing FAs, was transferred to vials for GC-MS analysis. The analyses were performed on 3 replicates of each skeleton. Three young corals and three old corals were collected in each site for each species.

GC-MS analyses were performed using a 6850 Agilent HP gas chromatograph connected to a 5975 Agilent HP quadrupole mass spectrometer. The injection port temperature was 280 °C. Analytes were separated by a HP-5 fused-silica capillary column (stationary phase poly [5% diphenyl/95% dimethyl] siloxane, 30 m, 0.25 mm i.d., 0.25 μm film thickness), with helium as carrier gas (at constant pressure, 33 cm s^−1^ linear velocity at 200 °C). Mass spectra were recorded under electron ionization (70 eV) at a frequency of 1 scan s^−1^ within the 12–600 m/z range (see Supplementary Fig. S1). The temperature of the column was increased from 50 °C up to 180 °C at 50 °C min^−1^, then from 180 °C up to 300 °C at 5 °C min^−1^. Methyl nonadecanoate was used as internal standard for the quantification of each FA, by assuming a unitary response factor.

### Statistical analysis of data

Fatty acid concentration was analyzed using permutational multivariate analysis of variance (PERMANOVA), which does not require homogeneity of variance or normal distributions^33^. Two PERMANOVA tests were run using euclidean distances among samples and 999 permutations in the software Primer®^34^, including the Monte Carlo correction for small sample size^35^. The Monte Carlo correction solves problems in non-parametric tests for small samples, because it estimates the p-value by taking a random sample from the reference set and studies its permutations^36^. The first Permanova model considered only the populations where all species were found and included the effects of the factors age (AG, two levels: young, old), population (PO, two levels: Palinuro and Pantelleria), species (SP, four levels: CCA, BEU, ACL, LPR), and their interactions. The second Permanova model considered only the species that were found in all populations and included the effects of the factors age (two levels: young, old), population (three levels: Calafuria, Palinuro, Pantelleria), species (three levels: CCA, BEU, LPR), and their interactions.

## Results

### Fatty acid concentration

**Figure 2:**
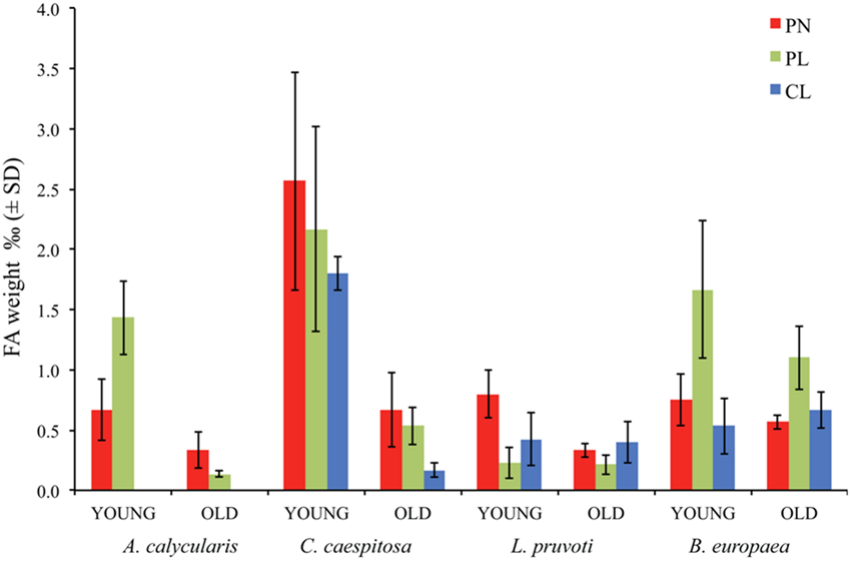
Intra-skeletal fatty acid concentrations. Concentration of intra-skeletal fatty acids in young and old *A*. *calycularis*, *C*. *caespitosa*, *L*. *pruvoti* and *B*. *europaea* skeletons collected along a latitudinal gradient of temperature in the Tyrrhenian Sea at Pantelleria island (PN), Palinuro (PL) and Calafuria (CL). *A*. *calycularis* was not found in CL.

A laboratory developed procedure allowing the detection of microgram of FAs was applied to overcome the difficulties due to the low concentration of intra-skeletal FAs. The FA concentration (as mass ‰) is reported in Table 1 and illustrated in Fig. 2. In both PERMANOVA models for FA concentration, the interaction terms SP × PO and SP × AG were significant (Table 2), and the pair-wise comparisons among levels of each factor were computed using these terms (see Supplementary Tables S1 and S2 for the first model and S3 and S4 for the second model). According to pair-wise comparisons of both models, young individuals had higher FA concentrations than old individuals, except in BEU where concentrations were homogeneous (see Supplementary Tables S1 and S3). CCA and ACL young skeletons showed a concentration of FA, 2.23 ± 0.74 (wt‰) and 1.05 ± 0.49 (wt‰) respectively, that is about 4.5 times higher than that in old samples, 0.46 ± 0.28 (wt‰) and 0.24 ± 0.15 (wt‰) respectively. In LPR young skeletons show a FA concentration equal to 0.48 ± 0.30 (wt‰) and 0.32 ± 0.13 (wt‰) in old skeleton and the concentration ratio between young and old skeletons is about 1.5. The FA content in BEU was 1.01 ± 0.64 (wt‰) and 0.78 ± 0.29 (wt‰) in young and the old skeletons, respectively, and significantly not different. A general trend of higher FA concentration in zooxanthellate [CCA = 1.29 ± 1.01 (wt‰) and BEU = 0.98 ± 0.55 (wt‰)] than in non-zooxanthellate corals [ACL = 0.64 ± 0.55 (wt‰) and LPR = 0.40 ± 0.24 (wt‰)] emerged, also confirmed by the pair-wise comparisons for the first model. The only exceptions were the homogeneous FA concentration in: 1) old ACL and BEU individuals; 2) BEU, ACL, and LPR individuals at Pantelleria (see Supplementary Tables S1 and S2). The same trend was confirmed by the second model. The only exceptions were the homogeneous FA concentrations in: 1) old CCA and LPR individuals, and 2) LPR and BEU at Pantelleria and Calafuria (see Supplementary Tables S3 and S4). No case was observed of a significantly higher FA concentration in a non-zooxanthellate coral relative to a zooxanthellate. According to pair-wise comparisons of both models, no clear trend related to the population was observed (see Supplementary Tables S1–S4). The analysis of the FAs on the single species at the three sites shows that the concentration of FAs moving from PN to CL is homogeneous (Fig. 2; see Supplementary Tables S2 and S4).

**Table 1:** Content of fatty acids (wt‰) extracted from *A*. *calycularis* (ACL), *C*. *caespitosa* (CCA), *L*. *pruvoti* (LPR) and *B*. *europaea* (BEU) skeletons collected along a latitudinal gradient of temperature in the Tyrrhenian Sea at Pantelleria island (PN), Palinuro (PL) and Calafuria (CL).

	ACL		CCA		LPR		BEU	
	(C, N-Z)		(C, Z)		(S, N-Z)		(S, Z)	
	*young*	*old*	*young*	*old*	*young*	*old*	*young*	*old*
PN	0.67 ± 0.25	0.33 ± 0.15	2.57 ± 0.91	0.67 ± 0.31	0.80 ± 0.20	0.33 ± 0.06	0.75 ± 0.21	0.57 ± 0.06
PL	1.43 ± 0.31	0.14 ± 0.03	2.17 ± 0.85	0.53 ± 0.15	0.23 ± 0.13	0.21 ± 0.08	1.67 ± 0.57	1.10 ± 0.26
CL	—	—	1.80 ± 0.14	0.17 ± 0.06	0.42 ± 0.22	0.40 ± 0.17	0.53 ± 0.23	0.67 ± 0.15
Age^a^	1.05 ± 0.49	0.24 ± 0.15	2.23 ± 0.74	0.46 ± 0.28	0.48 ± 0.30	0.32 ± 0.13	1.01 ± 0.64	0.78 ± 0.29
All^b^	0.64 ± 0.55		1.29 ± 1.05		0.40 ± 0.24		0.98 ± 0.55	

Three skeletons were analyzed for each species. ACL was not found in CL. C = Colonial, S = solitary, N-Z = Non-zooxanthellate, Z = Zooxanthellate. The standard deviation is reported.

^a^Average FA content in the skeletons from each species grouped in young and old age classes. ^b^Average FA content calculated considering for each species all the data from all the skeletons, independently from the age.

**Table 2:** Permutational multivariate analysis of variance (PERMANOVA) results for comparisons of FA concentration by population (PO), Species (SP), Age (AG), and their interactions.

Source	df	SS	MS	Pseudo-F	P(perm)	P(MC)
First model						
SP	3	8.10 × 10^−02^	2.70 × 10^−02^	17.968	0.001*	0.001*
PO	1	1.14 × 10^−03^	1.14 × 10^−03^	0.76166	0.401	0.402
AG	1	7.43 × 10^−02^	7.43 × 10^−02^	49.466	0.001*	0.001*
SP × PO	3	2.14 × 10^−02^	7.14 × 10^−03^	4.7526	0.007*	0.007*
SP × AG	3	4.23 × 10^−02^	1.41 × 10^−02^	9.3738	0.001*	0.002*
PO × AG	1	7.14 × 10^−04^	7.14 × 10^−04^	0.47525	0.512	0.498
SP × PO × AG	3	9.36 × 10^−03^	3.12 × 10^−03^	2.0759	0.114	0.107
Res	31	4.66 × 10^−02^	1.50 × 10^−03^			
Total	46	0.28122				
Second model						
SP	2	7.29 × 10^−02^	3.64 × 10^−02^	26.754	0.001*	0.001*
PO	2	1.03 × 10^−02^	5.17 × 10^−03^	3.7924	0.041*	0.027*
AG	1	6.24 × 10^−02^	6.24 × 10^−02^	45.819	0.001*	0.001*
SP × PO	4	2.62 × 10^−02^	6.54 × 10^−03^	4.8022	0.007*	0.003*
SP × AG	2	6.63 × 10^−02^	3.31 × 10^−02^	24.331	0.001*	0.001*
PO × AG	2	2.53 × 10^−03^	1.27 × 10^−03^	0.93014	0.413	0.395
SP × PO × AG	4	3.53 × 10^−03^	8.82 × 10^−04^	0.64777	0.621	0.636
Res	34	4.63 × 10^−02^	1.36 × 10^−03^			
Total	51	0.29474				

Tests were run using euclidean distances among samples and 999 permutations in the software Primer®, including the Montecarlo correction for small sample size. The first Permanova model considered only the populations where all species were found and included the effects of the factors AG (two levels: young, old), PO (two levels: Palinuro and Pantelleria), SP (four levels: *C*. *caespitosa*, *B*. *europaea*, *A*. *calycularis*, *L*. *pruvoti*), and their interactions. The second Permanova model considered only the species that were found in all populations and included the effects of the factors AG (two levels: young, old), PO (three levels: Calafuria, Palinuro, Pantelleria), SP (three levels: *C*. *caespitosa*, *B*. *europaea*, *L*. *pruvoti*), and their interactions. df degrees of freedom, SS sum of squares, MS mean squares, P(perm) significance, P(MC) significance after Montecarlo correction. Significant effects (p < 0.05) are indicated with an asterisk (*).

### Fatty acid composition

Table 3 lists the FA composition (relative distribution %) of the extracts. The detected chain length ranged from C14:0 to C18:0, C18:1 was also observed. Branched chain FAs were not detected in the extracts. In young coral skeletons C14:0, C15:0, C16:0, C17:0, C18:0 and C18:1 were observed, while old samples revealed only the presence of C16:0 and C18:0. However, missing FA composition data is probably due to values below detection limit (μg). In BEU also C14:0 and C18:1 were detected. Odd numbered FAs, C15:0 and C17:0, were detected only in ACL young samples. C16:0 was the most abundant FA in all the samples. C18:1 was detected only in CCA and BEU samples.

**Table 3:** Composition (relative distribution %) of the fatty acids extracted from *A*. *calycularis* (ACL), *C*. *caespitosa* (CCA), *L*. *pruvoti* (LPR) and *B*. *europaea* (BEU) skeletons collected along a latitudinal gradient of temperature in the Tyrrhenian Sea in Pantelleria island (PN), Palinuro (PL) and Calafuria (CL).

		**ACL** (C, N-Z)		**CCA** (C, Z)		**LPR** (S, N-Z)		**BEU** (S, Z)	
		*young*	*old*	*young*	*old*	*young*	*old*	*young*	*old*
PN	*C14:0*	3.9 ± 0.9	—	2.3 ± 0.4	—	—	—	—	6.1 ± 2.4
	*C15:0*	4.5 ± 2.7	—	—	—	—	—	—	—
	*C16:0*	49.3 ± 2.3	52.6 ± 2.9	61.6 ± 1.4	67.4 ± 1.9	56.3 ± 4.1	55.8 ± 2.0	51.9 ± 1.0	53.0 ± 2.0
	*C17:0*	4.6 ± 0.6	—	—	—	—	—	—	—
	*C18:1*	—	—	14.4 ± 3.9	—	—	—	9.1 ± 0.2	20.4 ± 0.6
	*C18:0*	37.7 ± 1.9	47.4 ± 1.7	21.7 ± 4.7	32.6 ± 0.7	43.7 ± 1.9	44.2 ± 0.9	38.1 ± 2.1	20.6 ± 1.1
PL	*C14:0*	—	—	3.3 ± 1.0	—	—	—	2.8 ± 0.4	4.9 ± 0.7
	*C15:0*	—	—	—	—	—	—	—	—
	*C16:0*	50.9 ± 2.8	55.4 ± 2.3	62.1 ± 2.7	69.6 ± 2.9	50.6 ± 2.8	50.8 ± 2.6	56.3 ± 1.0	61.2 ± 0.9
	*C17:0*	—	—	—	—	—	—	—	—
	*C18:1*	—	—	11.7 ± 1.2	—	—	—	9.7 ± 0.5	14.3 ± 0.3
	*C18:0*	49.1 ± 0.9	44.6 ± 0.6	22.9 ± 3.2	30.4 ± 1.3	49.4 ± 1.4	49.2 ± 0.9	30.4 ± 1.3	19.5 ± 1.8
CL	*C14:0*			—	—	—	—	1.9 ± 0.2	5.0 ± 0.8
	*C15:0*							—	—
	*C16:0*			51.5 ± 1.5	69.2 ± 0.4	52.4 ± 1.8	50.7 ± 1.6	47.4 ± 3.4	66.4 ± 0.2
	*C17:0*							—	—
	*C18:1*			14.6 ± 3.1	—	—	—	16.5 ± 0.9	—
	*C18:0*			19.5 ± 0.1	30.8 ± 0.8	47.6 ± 1.8	49.3 ± 1.3	33.3 ± 1.7	28.6 ± 0.6

The concentration of detected fatty acids is reported as fraction. The FA extracted from three skeletons for each species were analyzed. Samples of ACL were not found in CL. When the sum of the percentages is not 100 some not assigned FA was present. The standard deviation is reported. C = Colonial, S = solitary, N-Z = Non-zooxanthellate, Z = Zooxanthellate.

## Discussion

FA concentration and composition in coral soft tissues are useful indicators for studying coral nutrition strategies and the variation of nutrient and food ingestion. Moreover, FA reserves are good indicators of the resilience and trophic plasticity of a coral^37^. Despite the fact that the preservation of lipids in coral aragonite skeletons has been demonstrated^22^, no species specific studies have been carried out on them as potential ecological proxies. In approaching such research the dependence of FA content and composition on environmental conditions must be taken into account^2, 6, 8–10^. For this reason samples were collected from three sites environmentally different in temperature, irradiance and related parameters [e.g. ref. 31 and 32]. No clear trend related to the population living in the diverse environmental conditions was observed. The results on the concentration of FAs in coral skeletons indicate that young corals seem to entrap more FAs in their skeletons than old ones. A possible explanation of this observation could be that young corals are just less densely mineralized, such that the ratio of FAs to mineral is higher^38, 39^. This occurs regardless of the species or the site of collection. Coral skeletons are not subject to a re-precipitation process during their growth^28, 40^, thus materials entrapped in the skeleton in different ages are preserved, unless specific chemical processes (e.g. amino acid racemization) related to aging occur^41^. The fact that coral skeletal material can be altered by boring organisms or coralline crustose algae is a possibility that cannot be completely excluded^42^, however the use of oral regions of the skeleton and the extreme attention during sample selection makes it unlikely. Samples were taken trying to select only the skeleton that was recently deposited by the organism, both in young and old individuals. The higher concentration of FAs in skeletons of young individuals could be explained by the involvement of FAs in the biomineralization process^24, 40^. Several calcifying organisms, including corals, use lipidic vesicles to transport ions to the sites of mineralization^43^. Once these vesicles reach the mineralization site they release the ions. At this stage, some of the FAs, or lipids, of the vesicles could be incorporated in the growing skeleton. Given that crystalline occlusions are favored when impurities are entrapped during a rapid crystallization^44^ and since growth rate decreases with increasing age^31, 32^, more vesicle components (or residues) could be incorporated in the skeleton in young individuals, determining a higher concentration of FAs compared to old individuals. The same hypothesis could be applied to explain the higher FA concentration observed in colonial corals, compared to solitary ones, as the former have higher growth rates compared to the latter. For instance, *C*. *caespitosa* growth rate at Baia di Fiascherino (Gulf of La Spezia, 44°039N, 9°559E) is ~2.92 mg mm^−2^ yr^−1^ ^45^. Baia di Fiascherino is halfway between Genova and Calafuria, where *B*. *europaea* growth rates are respectively 1.09 and 2.86 mg mm^−2^ yr^−1^ ^29^. If we consider the average growth rate between these two locations (1.97 mg mm^−2^ yr^−1^), *C*. *caespitosa* growth rate is 39% higher than in *B*. *europaea*. Moreover, a trend of higher FA concentration in zooxanthellate than in non-zooxanthellate corals was also observed, perhaps because growth rates in symbiotic species tend to be higher than in non-symbiotic species. *L*. *pruvoti* growth rates at Genova and Calafuria are 1.08 and 1.26 mg mm^−2^ yr^−1^, respectively^30^, whose average (1.17 mg mm^−2^ yr^−1^) is 51% lower than in *B*. *europaea*. This is in agreement with the observation that some modern symbiotic reef corals can deposit aragonite up to an order of magnitude faster than their non-symbiotic counterparts^46^. An additional hypothesis could depend on their different trophic strategies. In fact, FA composition of total lipids of invertebrates depends on both FA biosynthesis pathway in their tissues and FA composition of food sources. In symbiotic coral species, the phototrophic supply is delivered by symbiotic dinoflagellates^47^, which allocate more than 90% of photosynthate to the host^48^, significantly contributing to the total FA content of corals^14, 49^. Based on the assumption that intra-skeletal FAs are representative of those in the soft tissue, the higher FA concentrations observed in this study in symbiotic versus non-symbiotic species could also depend on their different trophic strategy. A decreased content of saturated FAs was previously observed in non-symbiotic *Dendronephthya* species compared to symbiotic soft coral species, hypothesizing that this difference could depend on their different energy intake strategy^3^.

Pair-wise comparison among populations for FA concentrations in the three species resulted homogeneous (see Supplementary Table S4). Additional info can be obtained from the analysis of FA composition as a function of species, age and collection site. Zooxanthellae (algae) and the coral host (animal) contain different poly unsaturated fatty acids (PUFAs), which can serve as the markers of either symbiont lipids or host lipids^3, 4^. These specific FAs markers can be applied to confirm the exchange of PUFAs between symbionts and the host^50, 51^. Oleic (C18:1) was observed only in the zooxanthellate species^49^. This agrees with previous studies reporting the presence of C18:1 induced by zooxanthellae biosynthesis. Palmitic (C16:0) and stearic (C18:0) acids, the most abundant in all the investigated species, indicate omnivorous or carnivorous feeding modes^52^. Intra-skeletal odd-chain-length FAs found in this study are typically associated with bacteria^53^ which can actively metabolize organic matter in coral skeletons. However, previous studies of a zooxanthellae-bearing symbiotic anemone suggest that odd-chain FAs are also present in zooxanthellae^13^ and in coral tissue^12^, so their source is not clear.

## Conclusions

This research presents characterization of intra-skeletal FAs from Mediterranean corals having different age, growth and energy intake strategies. The results suggest that in the limits of the studied species, intra-skeletal FA composition and concentration may be used as proxies of level of organization and trophic strategy and eventually coral seniority.

## Electronic supplementary material

Supplementary Information
