# Supplementary material for: Ecological relevance of skeletal fatty acid concentration and composition in Mediterranean scleractinian corals
Source: Sci Rep. 2017 May 16;7:1929. doi: 10.1038/s41598-017-02034-2 (PMC5434035; doi:10.1038/s41598-017-02034-2)
Supplement: Supplementary file 1 — Supplementary Information [file 41598_2017_2034_MOESM1_ESM.pdf]

Supplementary Information

Ecological relevance of skeletal fatty acid concentration and composition in Mediterranean scleractinian corals

Chiara Samorì[1], Erik Caroselli[2], Fiorella Prada[2], Michela Reggi[1], Simona Fermani[1], Zvy Dubinsky[3], Stefano Goffredo[2, *], Giuseppe Falini[1, *]

[1]Department of Chemistry 'Giacomo Ciamician', University of Bologna, via Selmi 2, I-40126, Bologna, Italy. [2]Marine Science Group, Department of Biological, Geological and Environmental Sciences, University of Bologna, via Selmi 3, 40126 Bologna, Italy. [3]The Mina & Everard Goodman Faculty of Life Sciences, Bar-Ilan University, Ramat-Gan 5290002, Israel.

Figure 1 page SI2

Table SA page SI4

Table SB page SI5

Table SC page SI6

Table SD page SI7

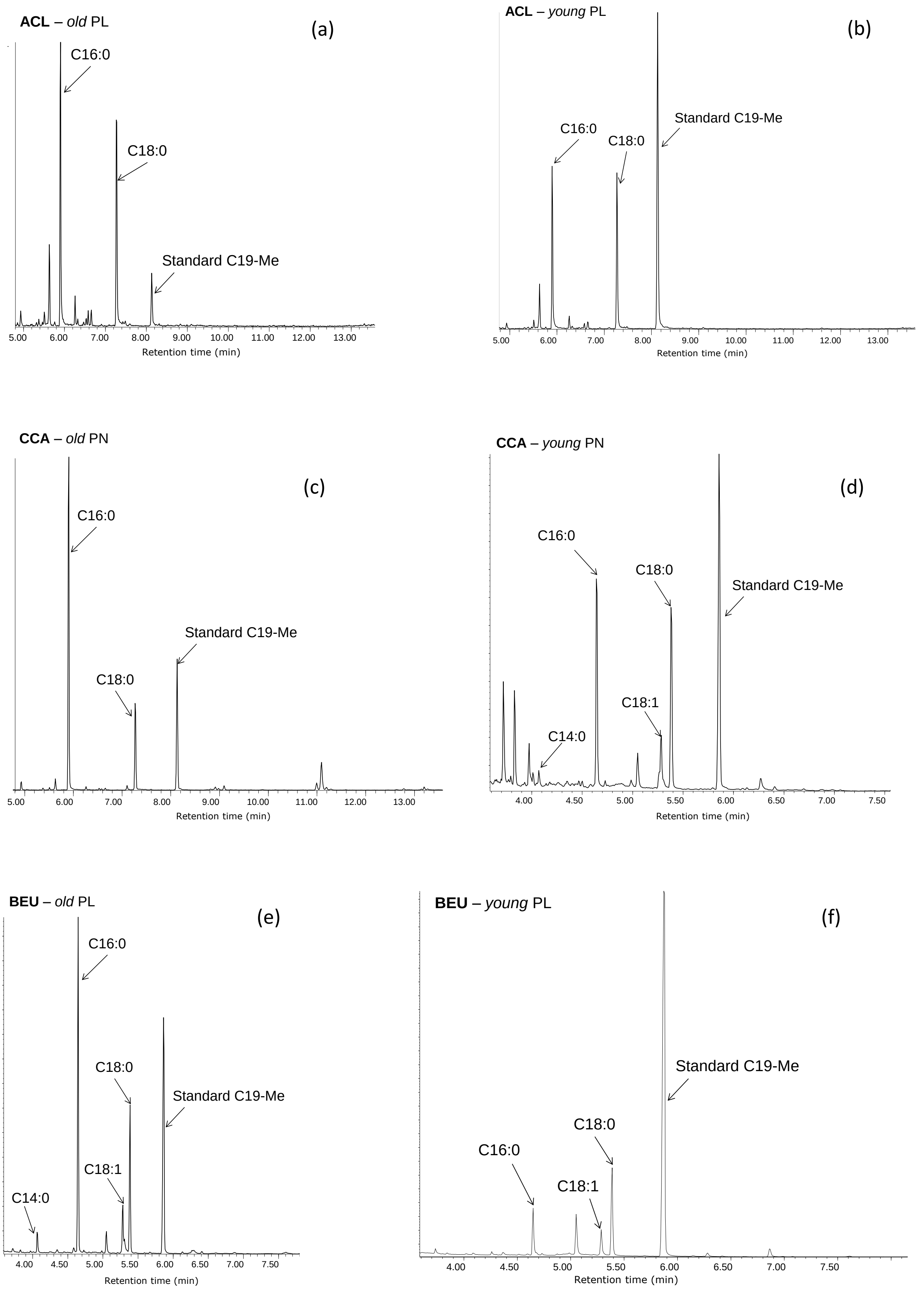
ACL – old PL
(a)
C16:0
C18:0
Standard C19-Me
5.00 6.00 7.00 8.00 9.00 10.00 11.00 12.00 13.00
Retention time (min)
ACL – young PL
(b)
Standard C19-Me
C16:0
C18:0
5.00 6.00 7.00 8.00 9.00 10.00 11.00 12.00 13.00
Retention time (min)
CCA – old PN
(c)
C16:0
Standard C19-Me
C18:0
5.00 6.00 7.00 8.00 9.00 10.00 11.00 12.00 13.00
Retention time (min)
CCA – young PN
(d)
C16:0
C18:0
Standard C19-Me
C18:1
C14:0
4.00 4.50 5.00 5.50 6.00 6.50 7.00 7.50
Retention time (min)
BEU – old PL
(e)
C16:0
C18:0
Standard C19-Me
C18:1
C14:0
4.00 4.50 5.00 5.50 6.00 6.50 7.00 7.50
Retention time (min)
BEU – young PL
(f)
Standard C19-Me
C18:0
C16:0
C18:1
4.00 4.50 5.00 5.50 6.00 6.50 7.00 7.50
Retention time (min)

SI2

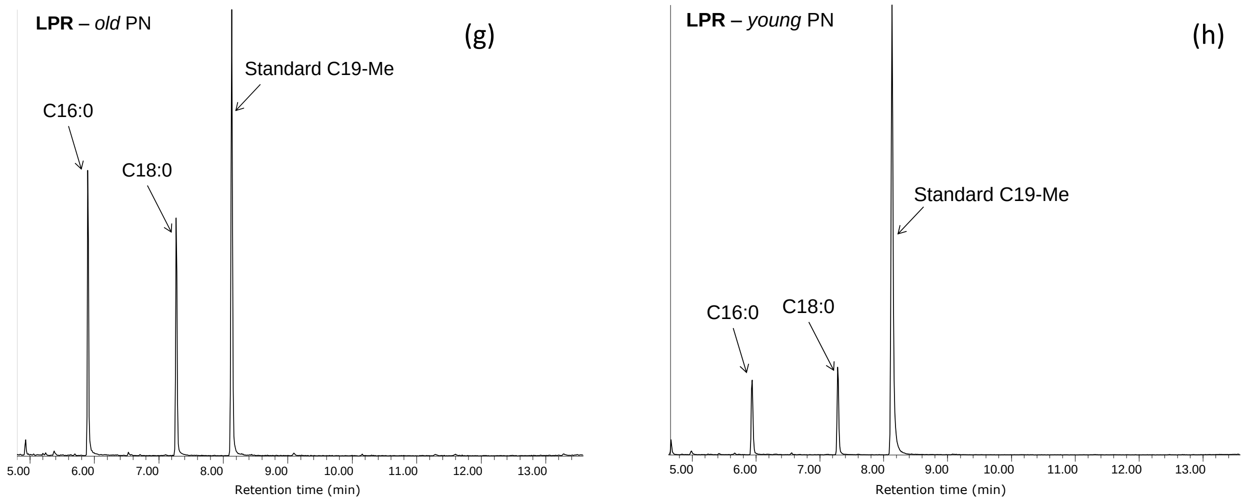

Fig. S1. Total Ion Current (TIC) chromatogram of fatty acids extracted from coral skeletons of old (a) and young (b) *Astroides calycularis*, old (c) and young (d) *Cladocora caespitosa*, old (e) and young (f) *Balanophyllia europaea*, and old (g) and young (h) *Leptopsammia pruvoti*, and analyzed by GC-MS.

Table S1 First Permanova model. Pair-wise comparisons among species and between ages for the FA concentration, based on the term SP x AG of the Permanova results. Significant effects ($p < 0.05$) are indicated with an asterisk. The column "details" reports a textual description of the trend of stiffness highlighted by the pair-wise comparisons in relation to the observed values reported in Table SA.

**Groups compared**	**Pseudo-t**	**P(perm)**	**P(MC)**	**Details**
Within young individuals				
CCA vs ACL	3.4948	0.007*	0.012*	FA concentration in CCA is higher than in ACL
CCA vs LPR	5.0716	0.002*	0.001*	FA concentration in CCA is higher than in LPR
CCA vs BEU	2.5724	0.039*	0.034*	FA concentration in CCA is higher than in BEU
ACL vs LPR	4.0329	0.010*	0.009*	FA concentration in ACL is higher than in LPR
ACL vs BEU	0.68237	0.495	0.494	FA concentration in ACL and BEU is homogeneous
LPR vs BEU	3.3502	0.021*	0.005*	FA concentration in BEU is higher than in LPR
Within old individuals				
CCA vs ACL	3.3716	0.011*	0.005*	FA concentration in CCA is higher than in ACL
CCA vs LPR	3.1825	0.010*	0.016*	FA concentration in CCA is higher than in LPR
CCA vs BEU	1.8543	0.115	0.097	FA concentration in CCA and BEU is homogeneous
ACL vs LPR	0.723	0.507	0.492	FA concentration in ACL and LPR is homogeneous
ACL vs BEU	6.6447	0.004*	0.001*	FA concentration in BEU is higher than in ACL
LPR vs BEU	6.7294	0.006*	0.001*	FA concentration in BEU is higher than in LPR
Within *Cladocora caespitosa* (CCA)				
Young vs old	4.7452	0.004*	0.001*	FA concentration in young individuals is higher than in old individuals
Within *Astroides calycularis* (ACL)				
Young vs old	6.6428	0.001*	0.001*	FA concentration in young individuals is higher than in old individuals
Within *Leptopsammia pruvoti* (LPR)				
Young vs old	3.246	0.014*	0.016*	FA concentration in young individuals is higher than in old individuals
Within *Balanophyllia europaea* (BEU)				
Young vs old	1.7695	0.136	0.126	FA concentration of young and old individuals is homogeneous

Table S2 First Permanova model. Pair-wise comparisons among species and between populations for the FA concentration, based on the term SP x PO of the Permanova results. Significant effects ($p < 0.05$) are indicated with an asterisk. The column "Details" reports a textual description of the trend of stiffness highlighted by the pair-wise comparisons in relation to the observed values reported in Table SB.

Groups compared	Pseudo-t	P(perm)	P(MC)	Details
Within Palinuro (PL)				
CCA vs ACL	2.1347	0.074	0.071	FA concentration in CCA and ACL is homogeneous
CCA vs LPR	4.464	0.002*	0.003*	FA concentration in CCA is higher than in LPR
CCA vs BEU	0.10815	0.929	0.907	FA concentration in CCA and BEU is homogeneous
ACL vs LPR	5.7408	0.003*	0.002*	FA concentration in ACL is higher than in LPR
ACL vs BEU	2.9692	0.015*	0.020*	FA concentration in BEU is higher than in ACL
LPR vs BEU	6.2511	0.002*	0.001*	FA concentration in BEU is higher than in LPR
Within Pantelleria (PN)				
CCA vs ACL	3.8618	0.005*	0.007*	FA concentration in CCA is higher than in ACL
CCA vs LPR	3.7123	0.011*	0.008*	FA concentration in CCA is higher than in LPR
CCA vs BEU	3.0157	0.014*	0.024*	FA concentration in CCA is higher than in BEU
ACL vs LPR	0.64051	0.529	0.509	FA concentration in ACL and LPR is homogeneous
ACL vs BEU	1.4422	0.200	0.207	FA concentration in ACL and BEU is homogeneous
LPR vs BEU	1.0648	0.312	0.311	FA concentration in LPR and BEU is homogeneous
Within *Cladocora caespitosa* (CCA)				
PL vs PN	0.71626	0.486	0.475	FA concentration in PL and PN is homogeneous
Within *Astroides calycularis* (ACL)				
PL vs PN	2.3229	0.055	0.045*	FA concentration in PL is higher than in PN
Within *Leptopsammia pruvoti* (LPR)				
PL vs PN	4.6887	0.005*	0.002*	FA concentration in PN is higher than in PL
Within *Balanophyllia europaea* (BEU)				
PL vs PN	3.4211	0.011*	0.006*	FA concentration in PL is higher than in PN

Table S3 Second Permanova model. Pair-wise comparisons among species and between ages for the FA concentration, based on the term SP x AG of the Permanova results. Significant effects ($p < 0.05$) are indicated with an asterisk. The column "Details" reports a textual description of the trend of stiffness highlighted by the pair-wise comparisons in relation to the observed values reported in Table SC.

Groups compared	Pseudo-t	P(perm)	P(MC)	Details
Within young individuals				
CCA vs LPR	6.2866	0.002*	0.001*	FA concentration in CCA is higher than in LPR
CCA vs BEU	3.7507	0.003*	0.009*	FA concentration in CCA is higher than in BEU
LPR vs BEU	3.3723	0.009*	0.008*	FA concentration in BEU is higher than in LPR
Within old individuals				
CCA vs LPR	1.82	0.105	0.088	FA concentration in CCA and LPR is homogeneous
CCA vs BEU	3.597	0.005*	0.006*	FA concentration in BEU is higher than in CCA
LPR vs BEU	6.5024	0.001*	0.001*	FA concentration in BEU is higher than in LPR
Within *Cladocora caespitosa* (CCA)				
Young vs old	6.3573	0.001*	0.001*	FA concentration in young individuals is higher than in old individuals
Within *Leptopsammia pruvoti* (LPR)				
Young vs old	2.3068	0.038*	0.044*	FA concentration in young individuals is higher than in old individuals
Within *Balanophyllia europaea* (BEU)				
Young vs old	1.3953	0.188	0.183	FA concentration of young and old individuals is homogeneous

Table S4 Second Permanova model. Pair-wise comparisons among species and among populations for the FA concentration, based on the term SP x PO of the Permanova results. Significant effects ($p < 0.05$) are indicated with an asterisk. The column "details" reports a textual description of the trend of stiffness highlighted by the pair-wise comparisons in relation to the observed values reported in Table SD.

**Groups compared**	**Pseudo-t**	**P(perm)**	**P(MC)**	**Details**
Within Palinuro (PL)				
CCA vs LPR	4.464	0.007*	0.004*	FA concentration in CCA is higher than in LPR
CCA vs BEU	0.10815	0.941	0.927	FA concentration in CCA and BEU is homogeneous
LPR vs BEU	6.2511	0.005*	0.001*	FA concentration in BEU is higher than in LPR
Within Pantelleria (PN)				
CCA vs LPR	3.7123	0.003*	0.006*	FA concentration in CCA is higher than in LPR
CCA vs BEU	3.0157	0.014*	0.016*	FA concentration in CCA is higher than in BEU
LPR vs BEU	1.0648	0.350	0.335	FA concentration in LPR and BEU is homogeneous
Within Calafuria (CL)				
CCA vs LPR	5.8033	0.005*	0.002*	FA concentration in CCA is higher than in LPR
CCA vs BEU	3.9037	0.011*	0.006*	FA concentration in CCA is higher than in BEU
LPR vs BEU	1.663	0.127	0.131	FA concentration in LPR and BEU is homogeneous
Within *Cladocora caespitosa* (CCA)				
PL vs PN	0.71626	0.508	0.487	FA concentration in PL and PN is homogeneous
PL vs CL	1.2849	0.257	0.232	FA concentration in PL and CL is homogeneous
PN vs CL	2.0064	0.072	0.091	FA concentration in PN and CL is homogeneous
Within *Leptopsammia pruvoti* (LPR)				
PL vs PN	4.6887	0.005*	0.003*	FA concentration in PN is higher than in PL
PL vs CL	2.1049	0.082	0.080	FA concentration in PL and CL is homogeneous
PN vs CL	1.5464	0.164	0.166	FA concentration in PN and CL is homogeneous
Within *Balanophyllia europaea* (BEU)				
PL vs PN	3.4211	0.011*	0.012*	FA concentration in PL is higher than in PN
PL vs CL	3.9581	0.008*	0.006*	FA concentration in PL is higher than in CL
PN vs CL	0.55664	0.604	0.576	FA concentration in PN and CL is homogeneous
